# Pneumocephalus and Seizure Post Cranioplasty: A Case Report

**DOI:** 10.7759/cureus.102003

**Published:** 2026-01-21

**Authors:** Tiago de Almeida Macruz, Gisele J Wakim, Livia Dahmen Rodrigues, Hudson Martins de Brito, Arthur V D Rezende

**Affiliations:** 1 Department of Anesthesiology, University of Miami, Miami, USA; 2 Department of Anesthesiology, University of Miami Miller School of Medicine/Jackson Memorial Hospital, Miami, USA; 3 Department of Medicine, Federal University of Ceará, Fortaleza, BRA; 4 Department of Medicine, Federal University of Pelotas, Pelotas, BRA

**Keywords:** anesthesia recovery period, brain injuries, cranioplasty, pneumocephalus, seizure, traumatic

## Abstract

Pneumocephalus is a condition characterized by the presence of air within the intracranial space and can be classified as either simple or tension. It is associated with various etiologies, ranging from infectious causes to craniofacial surgical procedures such as cranioplasty. We report the case of a 28-year-old male patient with no previous comorbidities who after sustaining a gunshot wound to the head, underwent a cranioplasty for facial reconstruction under general anesthesia. During the immediate postoperative period, the patient exhibited delayed awakening followed by a seizure, requiring reintubation and intensive care support. Imaging studies revealed the presence of simple pneumocephalus. Although pneumocephalus is a known complication of cranioplasty surgeries, this case emphasizes the importance of continuous clinical monitoring by the healthcare team throughout the surgical period, including the anesthetic recovery, in order to enable the early identification and management of signs and symptoms associated with potential adverse outcomes.

## Introduction

Pneumocephalus, also referred to as intracranial aerocele, is a condition defined by the presence of air within the intracranial space [1]. Although widely recognized today, the first documented case dates back to 1884, when Chiari described the condition post-mortem in a patient who died as a result of complications associated with ethmoiditis [2].

This phenomenon can affect individuals across all ages, including adults and children, and is typically classified according to the time of onset as either acute (<72 hours) or delayed (≥72 hours), and also based on location, it may be subarachnoid, epidural, subdural, intracerebral, or intraventricular [3]. In general, pneumocephalus is regarded as a complication that may arise from a broad spectrum of etiologies, including neoplasms, infections, craniofacial trauma, otorhinolaryngological procedures, head and neck surgeries, craniomaxillofacial operations, and idiopathic causes [1,4]. Notably, its occurrence may or may not be directly related to neurosurgical interventions.

Pneumocephalus can be classified as either simple or hypertensive. In general, it’s associated with additional neurological manifestations such as seizures, meningitis, severe headache, and altered levels of consciousness, all of which may adversely affect patient outcomes [5,6]. Hypertensive cases are considered neurosurgical emergencies due to the risk of permanent neurological damage and, in extreme situations, progression to brain death [7-9].

Among the neurosurgical procedures implicated, cranioplasty has been associated with the development of pneumocephalus as a postoperative complication, which may, in turn, lead to neurological events such as epileptic seizures [7].

Management of symptomatic pneumocephalus remains clinically challenging and necessitates a meticulous and individualized therapeutic approach to ensure optimal patient outcomes. In this context, the present report describes a clinical case of seizure associated with pneumocephalus following cranioplasty, and highlights the importance of continuous vigilance by surgical and anesthetic teams throughout the entire operative process, including the anesthetic recovery process.

## Case presentation

The patient was a 28-year-old man with no comorbidities or history of continuous medication use, who was initially admitted to the hospital after sustaining a gunshot wound to the head.

On his primary admission, he presented with obliteration of the left frontal bone and left orbit, as well as multifocal bifrontal contusions, with suspected injury to the left anterior cerebral artery (ACA). A bifrontal craniectomy was performed, along with placement of an external ventricular drain and tracheostomy. The ventricular drain was later removed, and the patient was decannulated for discharge.

Even though the first surgical intervention was performed, there were still bone fragments and the need to perform facial reconstruction on the patient. After two months, a new cranial computed tomography (CT) scan revealed extensive fractures involving the facial bones, left frontal bone, and orbit, with left herniation, as well as multiple retained ballistic fragments, primarily in the left frontal lobe. Therefore, given this scenario, a new surgical procedure was indicated by neurosurgery and maxillofacial surgery, aiming at bifrontal cranioplasty with a customized polyetheretherketone (PEEK) implant and orbital reconstruction. Neurologically, the patient responded to commands, could verbalize simple phrases, including motor responses in the upper limbs movement.

Surgery course and outcome

The cranioplasty was performed under general anesthesia with orotracheal intubation. For sedation and analgesia, the following medications were used: propofol, midazolam, fentanyl, and dexmedetomidine. The approach entailed a bicoronal incision, followed by subperiosteal dissection performed in collaboration with the maxillofacial surgery team using a Molt Periosteal Elevator #9 and electrocautery. The supraorbital rim and frontal bone were mobilized for visualization of the frontal sinus. Obstruction of the nasofrontal duct was identified. The cranial and facial reconstruction was completed without issues.

Immediately after surgery, flumazenil and naloxone were administered as the patient presented with delayed awakening. Approximately three minutes after administration, the patient was extubated in the operating room. The patient exhibited spontaneous eye opening, responded to simple commands, and had a good respiratory drive with normal tidal volume. However, shortly thereafter, while still in the post-anesthesia care unit (PACU), the patient developed a generalized tonic-clonic seizure with a sudden decline in consciousness level, along with ocular deviation, lasting approximately two minutes. Consequently, the patient was reintubated and managed with the use of anticonvulsant levetiracetam.

**Figure 1:**
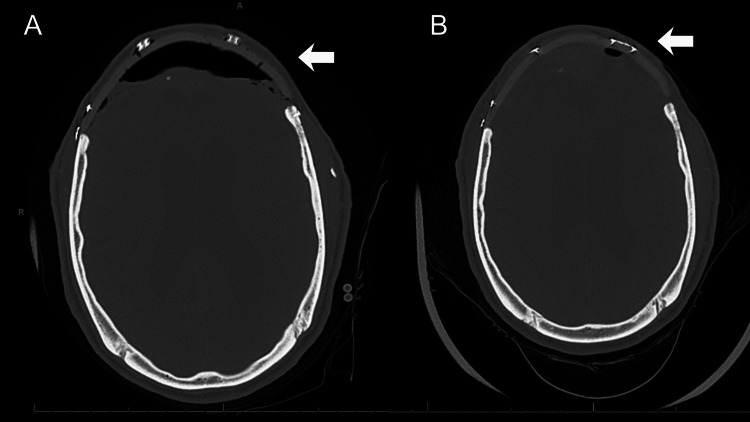
Cranial CT scan (A) Pneumocephalus identified after a postoperative decrease in the level of consciousness. (B) Follow-up imaging showing reduction of pneumocephalus 12 days post surgery.

After the seizure condition had stabilized, the patient was promptly taken for a cranial CT scan, which revealed postoperative mild pneumocephalus (Figure 1A). Additionally, interval development of mild dilation of the lateral ventricles was noted bilaterally, particularly in the right temporal horns, along with mild dilation of the third and fourth ventricles, findings suggestive of mild non-obstructive hydrocephalus.

The patient was subsequently admitted to the intensive care unit (ICU) for close neurological monitoring. He received medical management, including levetiracetam twice daily, and respiratory support for the following three days. During this period, he gradually returned to his neurological baseline, and serial CT scans confirmed a gradual resolution of the pneumocephalus. 

He remained in the ICU for neurological monitoring until the sixth postoperative day, when he was transferred to the ward for continuous follow-up. CT scans were performed during this period to observe the progression of the pneumocephalus. At no point did the patient exhibit hemodynamic or respiratory instability, nor were any other significant clinical or laboratory abnormalities identified. On the 12th postoperative day, CT imaging showed a significant reduction in intracranial air (Figure 1B). Given this radiological improvement and his stable clinical status, the patient was discharged after the 14th postoperative day. 

Notably, no further seizures occurred during hospitalization, and the patient remained on anticonvulsant therapy until his discharge. During follow-up in the outpatient clinic, there was no reported recurrence of seizures or other neurological symptoms related to pneumocephalus, so the event was interpreted as an acute symptomatic seizure in the immediate postoperative period. 

## Discussion

In this case, pneumocephalus was identified and attributed to the cranioplasty procedure, based on the temporal association between neurological deterioration and the presence of intracranial air, as well as the subsequent clinical improvement observed with its progressive resolution on CT imaging. The seizure was considered to be temporally and clinically associated with the presence of pneumocephalus in the immediate postoperative period. After clinical and radiological evaluation, no other contributing factors were identified to explain the postoperative neurological decline that led to reintubation and prolonged ICU stay. 

In the literature, this condition is considered a relatively common postoperative complication, especially in craniotomy surgeries, where its occurrence is reported in almost all cases [10,11]. Several intraoperative factors are known to increase the risk of developing pneumocephalus, including the use of nitrous oxide anesthesia, duration of surgery, head positioning, hyperventilation, and intraoperative osmotherapy, among others [12]. Knowledge of the risk factors is essential, as it allows, for example, the avoidance of nitrous oxide use in possible surgical reintervention. Although these factors are well recognized and preventive measures are often implemented, complete prevention of this complication cannot be guaranteed [10].

Furthermore, it is important to emphasize that, even though it is not the main hypothesis of the case discussed, the use of flumazenil, a benzodiazepine antagonist, may induce adverse drug reactions, including seizures [13]. In most cases, this reaction is not directly related to the drug itself, but rather to receptor antagonism following benzodiazepine intoxication [13,14].

A significant proportion of seizure cases occurs in patients who were already using pro-convulsant medications, such as tricyclic antidepressants, illicit drugs, had a prior history of seizures, or chronic benzodiazepine use [13,14]. These patients are, in the majority of cases, considered contraindicated for the use of flumazenil [13].

Nonetheless, it is not possible to directly associate the use of flumazenil with the onset of postoperative seizures as an additional factor beyond pneumocephalus itself as the causative agent. Furthermore, due to the decreased level of consciousness, the medical team was required to reintubate the patient. At the same time, in Huang et al.'s study (2018), it was observed that, among patients with postoperative pneumocephalus triggered by craniotomy in patients with brain tumours, reintubation was required in 2.9% of patients [15]. Although the patient in the current report did not have a brain tumor, these findings suggest that while uncommon, reintubation may be necessary due to intracranial aerocele.

Despite prior knowledge regarding pneumocephalus, this does not eliminate the need for close monitoring of cases already classified as simple, as they may evolve either asymptomatically or symptomatically, as observed in the patient described. Furthermore, intracranial air may fail to reabsorb spontaneously, requiring urgent neurosurgical intervention due to the risk of associated injuries.

Studies indicate that pneumocephalus undergoes spontaneous reabsorption in approximately 85% of cases [16]. This process may take three to five days in cases with minimal air accumulation, up to one week in mild cases, and two to three weeks when there is a significant amount of intracranial air [17,18]. 

In the current case, the patient was continuously monitored by the neurosurgery team throughout the hospital stay, with periodic cranial CT scans performed for follow-up, and a pre-scheduled date was established as a target for possible surgical intervention with air drainage if spontaneous reabsorption did not occur. However, he experienced clinical improvement that allowed for extubation three days after surgery, and significant radiological improvement and spontaneous resolution of the pneumocephalus 12 days after surgery. 

In a study conducted by Reasoner et al. (1994), pneumocephalus was observed in all patients who underwent supratentorial craniotomy within the first two postoperative days, with 66% classified as moderate to severe. In subsequent weeks, a progressive reduction in both volume and incidence was observed, with only 26.3% of patients still presenting pneumocephalus by the end of the third postoperative week [11].

Moreover, another relevant aspect analyzed in this case report was the importance of early imaging studies. Following the onset of neurological symptoms and decline in consciousness level after cranioplasty, prompt neuroimaging becomes essential, as acute signs or symptoms suggestive of potential brain injury warrant immediate cranial CT scanning, particularly in populations with known triggering etiologies. 

Although no specific study has yet directly addressed the optimal timing of imaging in cases of pneumocephalus, current evidence supports the value of early neuroimaging. In this context, a current consensus indicates that cranial CT imaging is highly sensitive for the detection of subarachnoid hemorrhage when performed within six hours of headache onset and interpreted by an experienced radiologist [19].

In addition to continuous monitoring, special attention is essential during the patient’s extubation and anesthetic recovery process. In the current case, although the patient initially showed an adequate response upon awakening from anesthesia, he rapidly developed a decreased level of consciousness and a seizure. This clinical deterioration required an immediate response from the medical team, which was decisive in achieving a favorable outcome.

In this context, it is also important to emphasize the need for close observation of the patient while in the PACU. Continuous monitoring should be maintained until the patient is clinically stable. Although no standardized duration exists for postoperative observation aimed specifically at identifying pneumocephalus, Florman et al. (2017) recommend a minimum of four hours of monitoring in the post-anesthesia care unit following an uncomplicated supratentorial craniotomy for tumor treatment [20]. Even though their study does not specifically refer to cranioplasty, this type of recommendation is particularly relevant in the context of the present case, in which early neurological deterioration occurred soon after extubation.

## Conclusions

Pneumocephalus remains a frequent complication following cranioplasty and may present with acute neurological manifestations, even when simple. This case illustrates that early postoperative neurological deterioration, including seizures and decreased level of consciousness, may occur in temporal association with intracranial air, and can require prompt airway management for support and intensive care. Early recognition, timely neuroimaging, and continuous postoperative monitoring, particularly during anesthetic recovery, are essential to ensure rapid intervention and favorable outcomes.

This report highlights the importance of maintaining a high suspicion for pneumocephalus in patients undergoing cranioplasty who develop acute neurological symptoms in the immediate postoperative period. In addition, during the management of the case and the preparation of this case report, it was possible to identify areas in which additional clarification may be beneficial, particularly with regard to the management of this type of case in anesthesia and anesthetic recovery. Further guidance, including considerations about recovery time, may support the medical team and contribute to safer clinical decision-making. 
